# Neurofibromin Deficiency and Extracellular Matrix Cooperate to Increase Transforming Potential through FAK-Dependent Signaling

**DOI:** 10.3390/cancers13102329

**Published:** 2021-05-12

**Authors:** Andrea Errico, Anna Stocco, Vincent M. Riccardi, Alberto Gambalunga, Franco Bassetto, Martina Grigatti, Amedeo Ferlosio, Gianluca Tadini, Debora Garozzo, Stefano Ferraresi, Andrea Trevisan, Sandra Giustini, Andrea Rasola, Federica Chiara

**Affiliations:** 1 Department of Surgery, Oncology and Gastroenterology, Veneto Institute of Oncology IOV-IRCCS, University of Padova, 35128 Padova, Italy; andrea.errico@univr.it; 2 Department of Biomedical Sciences, University of Padova, 35131 Padova, Italy; stocco.anna.7491@gmail.com (A.S.); andrea.rasola@unipd.it (A.R.); 3 The Neurofibromatosis Institute, La Crescenta, CA 91914, USA; vmriccardi@charter.net; 4 Department of Cardiac, Thoracic, Vascular Sciences and Public Health, University of Padova, 35128 Padova, Italy; alberto.gambalunga@unipd.it (A.G.); andrea.trevisan@unipd.it (A.T.); 5 Department of Neurosciences, University of Padova, 35128 Padova, Italy; franco.bassetto@unipd.it (F.B.); martina.grigatti@gmail.com (M.G.); 6 Department of Biomedicine and Prevention, Anatomic Pathology Institute, Tor Vergata University of Rome, 00133 Rome, Italy; ferlosio@med.uniroma2.it; 7 Center for Inherited Cutaneous Diseases, University of Milan, 20122 Milan, Italy; gtadinicmce@unimi.it; 8 Mediclinic Parkview Hospital, Dubai, United Arab Emirates; Debora.Garozzo@mediclinic.ae; 9 Ospedale S. Maria della Misericordia, Divisione di Neurochirurgia, 45100 Rovigo, Italy; stefano.ferraresi@aulss5.veneto.it; 10 Department of Clinical, Anesthesiologic and Cardio-Vascular Sciences, University of Sapienza, 00161 Rome, Italy; sandra.giustini@uniroma1.it

**Keywords:** neurofibromatosis type 1, tumor therapy, extracellular matrix, FAK, MEK inhibitor

## Abstract

**Simple Summary:**

Neurofibromatosis type 1 is a genetic disease that predisposes to tumors of the nervous system, primarily the neurofibroma. Plexiform neurofibromas (Pnfs) are of the greatest concern because of location, size, and frequent progression to malignancy. Although research is making great progress, the lack of in-depth understanding of the molecular mechanisms driving neoplastic progression results in the absence of prognostic indicators and therapeutic targets. We document that cell–cell cooperativity and the dynamics of the extracellular matrix play important roles in the growth and transformation of Pnf cells, directly through the cooperation of RAS and focal adhesion kinase (FAK) signaling. In turn, we found that treatment of Pnf cells with both MEK and FAK inhibitors is effective in abolishing the transforming ability of these cells.

**Abstract:**

Plexiform neurofibromas (Pnfs) are benign peripheral nerve sheath tumors that are major features of the human genetic syndrome, neurofibromatosis type 1 (NF1). Pnfs are derived from Schwann cells (SCs) undergoing loss of heterozygosity (LOH) at the *NF1* locus in an *NF1*^+/−^ milieu and thus are variably lacking in the key Ras-controlling protein, neurofibromin (Nfn). As these SCs are embedded in a dense desmoplastic milieu of stromal cells and abnormal extracellular matrix (ECM), cell–cell cooperativity (CCC) and the molecular microenvironment play essential roles in Pnf progression towards a malignant peripheral nerve sheath tumor (MPNST). The complexity of Pnf biology makes treatment challenging. The only approved drug, the MEK inhibitor Selumetinib, displays a variable and partial therapeutic response. Here, we explored ECM contributions to the growth of cells lacking Nfn. In a 3D in vitro culture, *NF1* loss sensitizes cells to signals from a Pnf-mimicking ECM through focal adhesion kinase (FAK) hyperactivation. This hyperactivation correlated with phosphorylation of the downstream effectors, Src, ERK, and AKT, and with colony formation. Expression of the GAP-related domain of Nfn only partially decreased activation of this signaling pathway and only slowed down 3D colony growth of cells lacking Nfn. However, combinatorial treatment with both the FAK inhibitor Defactinib (VS-6063) and Selumetinib (AZD6244) fully suppressed colony growth. These observations pave the way for a new combined therapeutic strategy simultaneously interfering with both intracellular signals and the interplay between the various tumor cells and the ECM.

## 1. Introduction

Neurofibromatosis type 1 (NF1), also known as von Recklinghausen disease, is an autosomal dominant disease caused by inactivating mutations of the *NF1* gene coding a 2818 amino acid protein, neurofibromin (Nfn).

The trademark of NF1 is the development of multiple benign peripheral nerve sheath tumors called neurofibromas. Neurofibromas are complex tumors originating from the peripheral nerve sheath, composed of Schwann cells (SCs) undergoing loss of heterozygosity (LOH) at the *NF1* locus in an *NF1*^+/−^ milieu composed by many other *NF1* haploinsufficient (HI) cells, including mast cells, macrophages and myofibroblasts [1]. The plexiform variety of neurofibroma (Pnf) can progress to the highly malignant sarcoma termed MPNST (malignant peripheral nerve sheath tumors), with a dismal prognosis [2]. A distinctive feature of the Pnf is a rigid extracellular matrix (ECM) structure, as myofibroblasts stimulated by TGF-β-secreting mast cells produce high quantities of insoluble proteins such as fibronectin and different types of collagens [3,4]. This causes both SC proliferation and deposition of an abnormal ECM. *NF1* HI cells display hyperactivation of Ras [5] that further increases when LOH at the *NF1* locus occurs [6]. Targeting MEK, a MAPKK downstream to Ras, has been the most successful treatment for neurofibromas up to now. Among MEK inhibitors, Selumetinib has been recently approved for Pnf treatment by the Food and Drug Administration [7,8,9,10]. However, variability in extent and duration of the therapeutic response to Selumetinib remains a major concern, making combinatorial therapies a possible and attractive development. Indeed, some NF1 children with inoperable Pnf benefited from long-term, dose-adjusted treatment with Selumetinib, but tumor shrinkage, albeit prolonged, remained partial (from 20% to 40%) [7,10]. Hence, a cogent approach would be a detailed pathogenic analysis of the molecular profile of Pnfs in order to identify target molecules that can lead to drugs enhancing chemosensitivity to Selumetinib.

Activation of Ras/Raf/Erk signaling in *NF1* HI SCs is necessary to make them more susceptible to proliferative signals provided by an *NF1* HI niche but not sufficient to induce full transformation of the cells [11]. The additional stimuli required to accomplish neoplastic progression remain poorly characterized and probably involve interactions with the ECM [12]. ECM homeostasis and dynamics are tightly regulated by soluble and insoluble components. These determine ECM biochemical and biomechanical features, which in turn influence tumor growth [13]. How lack of Nfn influences ECM–cell dynamic interactions and how ECM composition affects SC or fibroblast behavior are issues that remain unknown.

Among functional domains of the Nfn protein, a focal adhesion kinase (FAK)-binding region has been identified, and Nfn has been shown to interact with FAK [14]. FAK is a nonreceptor tyrosine kinase that associates with integrin receptors and participates in the transduction of ECM signals that control adhesion, movement, and proliferation of cells through activation of Src and PI3K/Akt cascades [15,16,17,18,19,20,21,22]. Therefore, the interaction between Nfn and FAK could be the central effector in the interplay between ECM and transformed cells, and a keystone in determining microenvironment contribution to Pnf onset and development [16,17,23]. Several lines of evidence highlight the importance of FAK in SC biology: absence of FAK severely impairs myelination [24], and neuregulin-erbB-FAK signaling is essential for migration during development and in cell spreading and guidance during nerve regeneration [24]. In fibroblasts, FAK signaling drives proliferation, secretion of metalloproteases (MMP) and fibrosis progression, and drugs or microRNAs inhibiting FAK/Akt signaling have displayed antineoplastic effects [25,26].

In breast cancer models, deregulated activation of FAK promotes tumor progression and metastasis by affecting the cross-talk between cancer and stromal cells and by sensitizing tumor cells to ECM biochemical and mechano-signals, with profound effects on their genomic landscape [27]. Pharmacological inhibition of FAK enhances chemosensitivity in taxane-resistant cells [28,29,30,31,32]. In pancreatic adenocarcinoma, where desmoplastic stroma functions as a barrier to T cell infiltration and to chemotherapy, inhibition of hyperactive FAK by Defactinib (VS-6063) reduces stromal density and the immunosuppressive features of the microenvironment [33,34,35].

In the present work, we assess the hypothesis that neoplastic transformation of neurofibroma SC requires both growth-factor signaling and matrix signaling contributed by FAK.

We find that the growth of cells lacking Nfn in a 3D system in which they were fully embedded by a Collagen/Fibronectin Matrigel mimicking the extracellular matrix of Pnfs is a result of the cooperation between Nfn loss and FAK kinase. This observation may open a novel pharmacological option for Pnf treatment, that is, by enhancing the response to Selumetinib (AZD6244) with the use of FAK inhibitors.

## 2. Material and Methods

### 2.1. Reagents

Rabbit anti-neurofibromin polyclonal antibodies were purchased from NOVUS Biological (NB100-418, Cambridge, UK). Actin (C-11) (sc-1615) from Santa Cruz Biotechnology (Heidelberg, Germany), mouse anti-Akt (#2920, 40D4), rabbit anti-phospho-Akt (S473, #9271), rabbit anti-Src (Y416) anti-GAPDH (#2118, Cell Signaling), rabbit anti-phospho-p44/42 ERK (#9101), anti-phospho-FAK (Y925 #3284), anti-FAK (#3285) were purchased from (Cell Signaling Technology, Danvers, MA, USA), anti-phospho-FAK (Y397) (44-624G, Invitrogen, Carlsbad, CA, USA). Laminin (Novus Biologicals NBP1-78301), Fibronectin (Sigma-Aldrich, Milano, Italy), Collagen I (Sigma-Aldrich, C8919-20ML) were used for plastic coating; Matrigel (BD Biosciences, Franklin Lakes, NJ, USA) growth factors reduced (GFR) for 3D in vitro cultures, h-EGF, PDGF-BB growth factors (Thermo Fisher Scientific Recombinant Proteins, Milano, Italy). Furthermore, 3-iso-butyl-L-methylxanthine (IBMX; Sigma-Aldrich), β1-heregulin (Sigma-Aldrich), forskolin and insulin (Sigma-Aldrich), anti-mouse IgG (whole molecule) were used for cell maintenance and Western blotting; Peroxidase Conjugate (Sigma-Aldrich), ECL anti-mouse/rabbit IgG, Horseradish Peroxidase Linked Species–Specific Antibody (GE Healthcare, Milano, Italy). Alexa Fluor^®^ 647 rabbit monoclonal (EP1576Y) to S100 beta (Abcam, Cambridge, MA, USA), Mounting Medium With DAPI-Aqueous, Fluoroshield (ab104139), Defactinib (VS-6063, PF-04554878 Selleckchem, Houston, TX, USA) is a FAK inhibitor, Y15, is a small molecule FAK scaffolding inhibitor that was shown to decrease cancer growth in vitro and in vivo [29]. Selumetinib (AZD6244, Selleckchem) is a potent anti-MEK inhibitor that has been shown to induce plexiform neurofibroma shrinkage in Phase I/II trials [10].

### 2.2. Cell Cultures

Mouse embryonic fibroblasts (MEFs) cultured in adherent conditions were maintained and grown in DMEM (Dulbecco’s modified eagle medium, high glucose with sodium pyruvate and L-glutamine, EuroClone, Milano, Italy). To this culture media, fetal bovine serum, FBS (10% *v*/*v*, fetal bovine serum, EuroClone) and 5 mM L-glutamine (L-glutamine solution 200 mM, EuroClone) were added; penicillin (2 mg/mL) and streptomycin (2 mg/mL) (penicillin/streptomycin solution 100×, EuroClone). Flasks/Petri dishes were maintained in an incubator at 37 °C in a humidified atmosphere with 5% of CO_2_. When required plastic dishes were coated with Collagen I (400 μL of Sigma C8919-20ML in 4 mL of gelatin), Laminin (5 μg/mL), or Fibronectin (10 μg/mL)

SCs ipNF 04.4 and ipNF 97.4 were cultured according to the M. Wallace protocol [36]. Briefly, we coat a 10 cm culture dish or flask with laminin (5 mL of 10 ug/mL Laminin) in 1×PBS (phosphate-buffered saline) onto a 10 cm culture dish, keep at 37 degrees for 1 h. Draw off the Laminin and rinse the plate three times with 5 mL of 1 × PBS. Collagen I can be substituted for Laminin: 400 uL of Sigma C8919-20ML in 4 mL of 1 × PBS, 2 h at 37 degrees, remove Collagen, rinse plate once with 5 mL 1 × PBS. Cells were stably transfected with Lipofectamine following manufacturer instructions (Thermo Fisher Scientific) and maintained in a selective medium 72 h post transfection in presence of G418, Geneticin (Thermo Fisher Scientific).

### 2.3. Isolation of SCs from Neurofibromas

Schwann and myofibroblast cells were isolated from tumor specimens according to Eduard Serra Protocol [36]. In brief, tumor pieces were shredded and then digested in DMEM, 10% FCS (Gibco BRL, Paisley, UK), 500 U/mL penicillin/streptomycin (Sigma-Aldrich), 160 U/mL collagenase type 1 (Sigma-Aldrich), and 1 U/mL dispase grade 1 (Sigma D4818). Following an incubation period of 20 h at 37 °C and 10% CO2 in this buffer, samples were further melted by pipetting with a narrowed Pasteur pipette. The suspension was moved to a 50 mL Falcon tube with DMEM with 10% FCS, centrifuged at 3000× *g* for 10 min, and suspended in a fresh buffer composed of DMEM, 10% FCS, 500 U/mL penicillin/streptomycin, 0.5 mM 3-iso-butyl-L-methylxanthine (IBMX; Sigma-Aldrich), 10 nM β1-heregulin (Peprotech, #100-03, London, UK), 0.5 µM forskolin and 2.5 µg/mL insulin (Sigma-Aldrich). Cells were seeded at a density of 25 000 cells/cm^2^ onto six-well plates (Falcon, Milville, NJ, USA), precoated with 1 mg/mL poly-L-lysine (Sigma-Aldrich) and 4 µg/mL natural mouse Laminin (Sigma-Aldrich). Cultures were incubated in a humidified atmosphere at 37 °C and 10% CO2. The medium was changed twice a week and cells were passed when cultures were confluent (usually after 5–7 days).

### 2.4. The 3D Culture In Vitro

Cells were fully embedded in Matrigel and maintained in standard 48-well plates. Culture wells were gently detached, counted in a Burker chamber, resuspended in a small volume of medium (50 μL/well with or without ECM proteins when required), and mixed with ~250 μL of Matrigel/10.000 cells/well (BD Biosciences). Matrigel is kept at 10 °C to avoid polymerization. After gelation at 37 °C for 20 min, 250 μL medium was added with appropriate supplements. It was exchanged every 48 h by gently removing from the top and adding 250 μL. To count colonies, we fixed and stained cells by using 0.2% crystal violet in 2% of ethanol: the medium must be removed before using the dye. After the staining phase, we performed different washes with variable volumes of PBS in order to remove as much as possible the dye in the medium that was incorporated by the cells. Then, we collected some images of the plate by using an EPSON scanner. The cell quantification has been performed by using Image J software.

### 2.5. Cell Lysates and Western Immunoblot Analysis

Cultured cells on 10 cm plates were lysed with 0.5 mL modified RIPA lysis buffer15; lysates were diluted on 0.5 mL HNTG buffer (50 mM HEPES pH 7.4, 150 mM NaCl, 0.1% Triton X-100 and 10% glycerol). For immunoprecipitations, lysates were incubated with agarose-Protein A beads at 4 °C overnight. The day after lysates were cleared by mild centrifugation and washed four times with the lysis buffer. As negative control lysates were incubated on conjugated beads in the absence of primary antibodies. Samples were then separated in reducing conditions on SDS-polyacrylamide gels and transferred onto Hybond-C Extra membranes (Amersham, Little Chalfont, UK). Primary antibodies were incubated overnight at 4 °C, and horseradish peroxidase-conjugated secondary antibodies were added for 1 h, allowing chemiluminescence detection (Amersham). Band densitometric analysis was performed using ImageJ image processing program.

### 2.6. Immunofluorescence Microscopy

The day before the experiment, cells were seeded on microscope slides coated with Collagen I/Laminin. On the day of the experiment, cells were washed in PBS (0.01 M), fixed with 2% paraformaldehyde. For the staining with antibodies, cells were then incubated for 3 h at 4 °C and in a wet and dark chamber with the primary antibodies at the concentration of 0.2 μg/mL, then washed and incubated for 30 min in the same conditions with the secondary antibody. The DAPI staining required 5 min of incubation at room temperature (300 nM DAPI-Aqueous, Fluoroshield (ab104139)). Sample analyses were conducted on a Leica DM-IRB fluorescence microscope equipped with a Hamamatsu C4742-95 digital camera, and images were stored with the use of the Image ProPlus v 7.0 software.

### 2.7. Statistical Analysis

Collected data were statistically analyzed by parametric Student’s *t*-test. In all histograms, mean ± S.D. (standard deviation of the mean) values were shown. *p*-values 0.01 were considered to be statistically significant.

## 3. Results

### 3.1. Neurofibromin Deficiency Affects Phosphorylation Kinetics of ERK and FAK under PDGF-BB Stimulation

**Figure 1:**
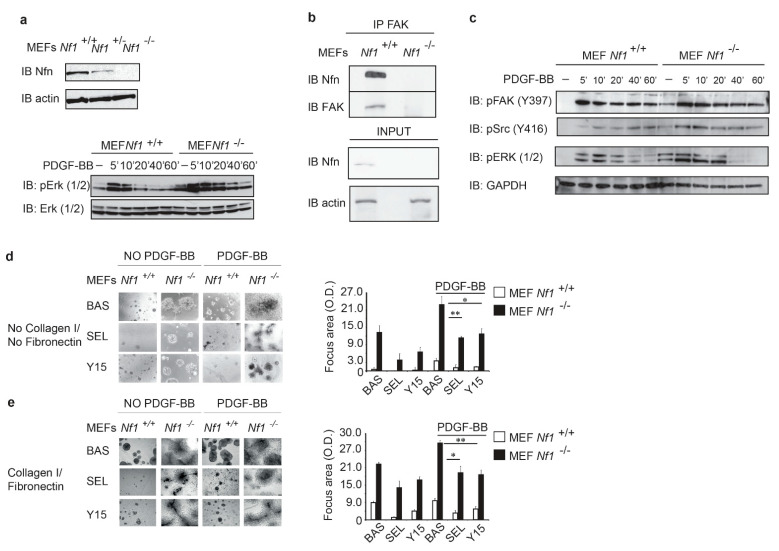
Neurofibromin loss sensitizes MEF cells to growth factor and matrix stimulation: (**a**) MEF cells isolated and immortalized from wild-type and knockout mice for *Nf1* gene were analyzed by Western blotting with anti-neurofibromin (Nfn) antibodies; (**b**) focal adhesion kinase FAK binds to Nfn. FAK was immunoprecipitated (IP) from cell lysates of wild-type and knockout MEFs (*Nf1*^+/+^, *Nf1*^−/−^); following SDS-PAGE electrophoresis, co-immunoprecipitated Nfn were assayed by Western blotting analysis (IB) with anti-FAK and anti-Nfn antibodies. Total protein loaded on the IP is also shown as INPUT ((**b**), lower panel) and examined by anti-Nfn and anti-actin antibodies; (**c**) transient phosphorylation kinetics of MEFs stimulated with PDGF-BB (10 ng/mL), [38,39] for increasing time periods. Following SDS-PAGE, cell lysates were incubated with anti-phospho-Erk (1/2) (T202/Y204, T185/Y187) MAP kinase, phospho-FAK (Y397) and phospho-Src (Y416) antibodies and anti-GAPDH; (**d**,**e**) colony formation assay of MEFs fully embedded by low growth factor containing Matrigel in absence (upper panel) or presence (lower panel) of insoluble matrix proteins Collagen I/Fibronectin. Cells were seeded in the Matrigel and 24 h later treated or not with PDGF-BB ligand for 8 days (representative microscope pictures (10×)). Where indicated, MEF cells were additionally treated with FAK and MEK inhibitors named Y10 (0.62 μM) and Selumetinib (6.62 μM), respectively; the area of colonies was calculated in five fields for each well, and mean value and SD calculated and plotted in the right histograms. *n* = 8; * *p* < 0.05 **; *p* < 0.001 by Student’s *t*-test.

In order to comprehend the ECM role in signaling modulation of *NF1* DI SCs, we used MEF cells isolated from wild type and *Nf1*^−/−^ mice [37] as a preliminary experimental system (*Nf1*^+/+^ and *Nf1*^−/−^ MEFs; Figure 1a). Expectedly [5], *Nf1*^−/−^ MEFs displayed hyperactivation of ERK1/2 following growth factor stimulation. Indeed, PDGF-BB stimulation [38,39] induced a faster, more prolonged, and higher ERK1/2 phosphorylation in MEFs when *Nf1* was absent (Figure 1a). To investigate whether the lack of Nfn sensitizes cells to ECM signaling, we studied FAK activation by assessing its auto-phosphorylation at tyrosine 397 (Y397) [15,40]. First, we found a direct FAK/Nfn interaction in wild-type MEFs (Figure 1b), where Y397 showed transient phosphorylation kinetics overlapping that of ERK under PDGF-BB stimulation. FAK activation was higher in *Nf1*-deficient MEFs, where Y397 phosphorylation was constitutive and further enhanced by PDGF-BB treatment (Figure 1c). Y397 phosphorylation creates a high-affinity binding site for the SH2 domain of Src, leading to its recruitment and activation [15]. Therefore, we assessed Src phosphorylation kinetics following PDGF-BB treatment, finding that in *Nf1*^−/−^ MEFs Src was active in basal condition and its phosphorylation increased after PDGF-BB stimulation (Figure 1c). These observations suggest the existence of a signaling cross-talk between Nfn loss and FAK, enhanced by growth factor binding.

### 3.2. Cell Transformation Induced by Neurofibromin Deficiency Is Increased by PDGF-BB and Further Enhanced by Collagen/Fibronectin

We developed a variant of the colony formation assay, which is a well-known technique used to evaluate cell tumorigenicity by measuring growth in the absence of substrate binding. In our modified approach, cells are fully embedded in a Matrigel containing low levels of growth factors. This matrix can be enriched with Collagen/Fibronectin for inducing integrin clustering and the ensuing FAK activation. With this approach, we observed that *Nf1*^−/−^ MEFs had a high ability to form colonies, and this was increased under PDGF-BB stimulation (Figure 1d) and further potentiated when we added a mixture of Collagen/Fibronectin to the Matrigel matrix (Figure 1e). These results suggest a cooperative contribution of pathways elicited by *Nf1* loss and by FAK activation in fostering neoplastic growth. Consistently, *Nf1*^−/−^ MEF colony development was sensitive to inhibitors of MEK (Selumetinib, AZD6244) and FAK (Y15) [41]. Of note, neither FAK nor MEK inhibition fully abrogated colony formation (Figure 1d,e), suggesting the coexistence of multiple molecular pathways cooperating in cell transformation.

### 3.3. Oncogenic Signaling Mediated by Both RAS and FAK Signaling Sustains Transformation in Nfn-Deficient Cells

**Figure 2:**
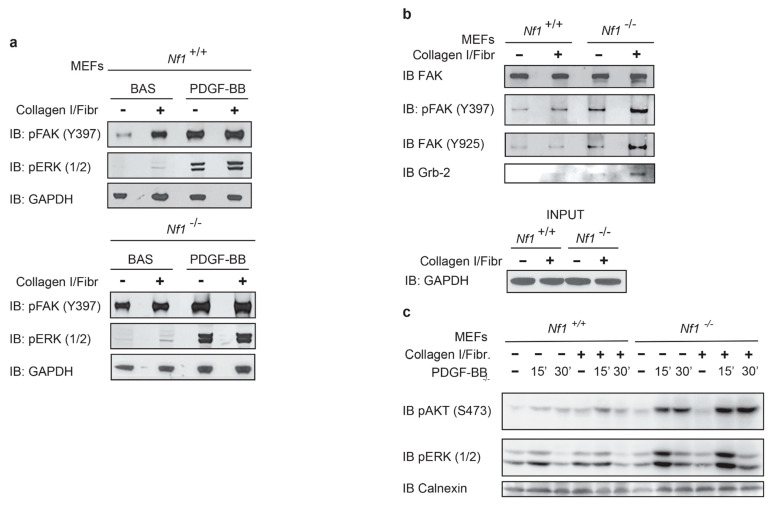
Extracellular matrix boosts ERK and AKT phosphorylation through FAK phosphorylation: (**a**) cell treatment with both PDGF-BB ligand and Collagen/Fibronectin activate FAK and ERK phosphorylation. Maximal ERK phosphorylation is reached following combinatorial treatment of PDGF-BB and Collagen/Fibronectin. *Nf1*^+/+^ MEF cells (upper panel) and *Nf1*^−/−^ MEF cells (lower panel) were starved, gently detached, and seeded for 24 h on plastic wells coated with gelatin alone or Collagen/Fibronectin. Cell lysates were run in SDS-PAGE electrophoresis, transferred on nitrocellulose, and analyzed by Western blotting with phospho-FAK (Y397), phospho-ERK (1/2), and anti-GAPDH for protein loading assessment. *n* = 5; (**b**) active FAK is a scaffold protein for the growth factor receptor-bound protein 2, Grb-2. After 24 h of culture on plastic wells coated with gelatin alone or Collagen/Fibronectin, cells were lysed and subjected to immunoprecipitation of FAK with anti-FAK antibody. Subsequent Western blotting analysis of immunoprecipitated complexes was performed by FAK Y925 phospho-specific antibody, phospho-ERK (1/2), and anti-Grb-2 antibodies. Anti-GAPDH was instrumental to monitor cell lysate loading in the INPUT (lower panel). *n* = 3; (**c**) Western blotting analysis of lysates of MEFs stimulated with PDGF-BB (10 ng/mL) alone or in combination with Collagen/Fibronectin with phospho-AKT and phospho-ERK antibodies. Calnexin was instrumental to monitor cell lysate loading. *n* = 3. (Figure S1).

We then investigated the phosphorylation state of ERK and FAK following PDGF-BB treatment and Collagen I/Fibronectin stimulation. We observed that maximal phosphorylation of both ERK and FAK was reached following concomitant growth factor/ECM stimulation in Nfn-deficient MEFs, in further agreement with a cooperative role played by both stimuli (Figure 2a). FAK is strongly activated in *Nf1*^−/−^ cells at both Y397 and at residue Y925, which is instrumental in recruiting Grb-2 (Figure 2b) and eliciting downstream induction of Ras signaling [18,19,20,21,22,42,43]. Furthermore, in the same conditions of maximal FAK activation, we also detected a high induction of Akt phosphorylation (Figure 2c).

**Figure 3:**
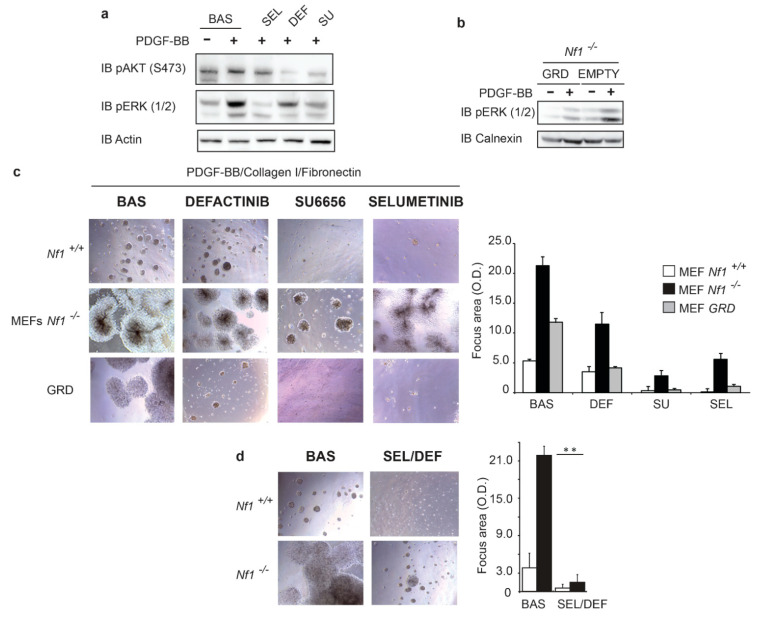
FAK-dependent activation of Ras and AKT molecular pathways trigger transforming ability in the *Nf1*-deficient cells: (**a**) Western blotting analysis with anti-phospho-ERK, anti-phospho-AKT, and anti-Calnexin antibodies of cell lysate of colonies of *Nf1*^−/^^−^ MEF cultured in Matrigel enriched with growth factor/Collagen/Fibronectin. Cells were treated with different inhibitors: Selumetinib, Defactinib, and SU6656, inhibiting MEK, FAK, and Src, respectively; (**b**) Western blotting analysis performed with anti-phospho-ERK (1/2) of cell lysates constitutively transfected with the catalytic domain of the Ras-Gap Nf1 and treated or not with PDGF-BB; (**c**) colony formation assay with *Nf1*^+/+^ MEF and *Nf1*^−/^^−^ MEF transfected with *Nf1*-GRD or empty vector pcDNA3, fully embedded by low growth factor containing Matrigel in presence of both insoluble matrix proteins Collagen I/Fibronectin and PDGF-BB ligand. Cells were seeded in the Matrigel and treated with PDGF-BB ligand. Where indicated, MEFs cells were additionally treated with Defactinib (6.25 μM), Selumetinib (0.62 μM), and SU6656 (2.5 μM) for 8 days. The area of colonies was calculated in five fields for each well and the mean value and SD were calculated and plotted in the right histograms. *n* = 6; (**d**) colony formation assay with *Nf1*^−/^^−^ MEF fully embedded by a low growth factor containing Matrigel in presence of both insoluble extracellular matrix proteins as Collagen I/Fibronectin and PDGF-BB ligand. Cells were treated with a combination of the FAK and MEK inhibitors Defactinib (6.25 μM) and Selumetinib (0.62 μM) for 8 days. The area of colonies was calculated in 5 fields for each well and the mean value and SD were calculated and plotted in the right histograms. Most representative experiment of *n* = 3; ** *p* < 0.001 by Student’s *t*-test.

Cell treatment with the FAK inhibitor Defactinib (vs-6063), a very promising molecule well tolerated by patients [31], along with the Src inhibitor SU6656 and with the MEK inhibitor Selumetinib, partially inhibited both AKT and ERK phosphorylation (Figure 3a). In accord with a crucial role played by these pathways in tumorigenicity of *Nf1*^−/−^ MEF exposed to PDGF-BB and Collagen/Fibronectin, colony growth was significantly but partially reduced by treatment with Defactinib, SU6656, or Selumetinib, as well as by expression of Nf1-GRD, the catalytic domain of Nfn, which inhibited ERK1/2 hyperactivation (Figure 3b). Notably, treatment of Nf1-GRD-expressing MEFs with kinase inhibitors fully abrogated colony formation (Figure 3c, lower row), strongly indicating the synergistic contribution of multiple signaling pathways in fostering transformation. Taken together, these results indicate that in Nfn-deficient cells, FAK boosts both Ras and AKT activity in a growth factor/ECM-dependent manner. Consistently, the concomitant pharmacological interference of both Ras and FAK signaling pathways in *Nf1*^−/^^−^ MEFs with Defactinib and Selumetinib completely abrogated colony growth (Figure 3d).

### 3.4. Neurofibromin Deficiency and FAK Cooperate to Fuel Tumorigenicity of SCs from PNs

**Figure 4:**
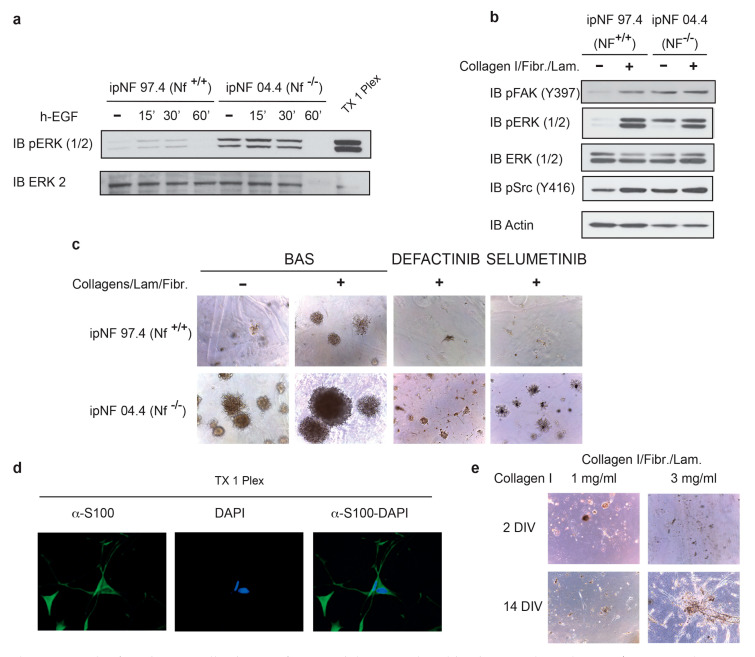
In plexiform human cells, the transforming ability is mediated by the FAK-dependent Ras/AKT cascades: (**a**) phosphorylation kinetics of ERK in cells isolated from human plexiform neurofibromas from individuals affected by NF1 (ipNF 04.4) and nerves of healthy people (ipNF 97.4) and immortalized [36]. Starved cells were stimulated for different time-period with h-EGF; (**b**) Western blotting analysis by phospho-specific antibodies and actin reveals the sensitivity of the FAK, ERK, and Src phosphorylation to ECM stimuli. Maximal phosphorylation was observed in cells lacking *NF1* (ipNF 04.4). Representative experiment of *n* = 3; (**c**) colony formation assay of fully embedded cells by low growth factor containing Matrigel in absence (upper panel) or presence (lower panel) of insoluble matrix proteins Collagen I/Laminin/Fibronectin. Cells were seeded in the Matrigel enriched with h-EGF ligand plus Collagens/Fibronectin/Laminin for 8 days (representative microscope pictures (10×)). Where indicated, MEFs cells were additionally treated with FAK and MEK inhibitors named Y10 (0.62 μM) and Selumetinib (6.62 μM), respectively, they were added to the cell culture after 48 h from the plating; the area of colonies was calculated in five fields for each well and mean value and SD calculated and plotted in the right histograms. *n* = 8; (**d**) immunofluorescence staining of primary cells (named PX 1) from human plexiform neurofibroma with an AlexaFluor-conjugated anti-S100 antibody and DAPI marking the nucleus. The merge of both signals is also shown. *n* = 3; (**e**) representative pictures of primary SC cells, PX 1 cells at 2 and 14 days in culture (2 DIV: 2 day in vitro) fully embedded in Matrigel enriched by Laminin/Fibronectin/Collagen I, where Collagen I was added at different doses (1 mg/mL and 3 mg/mL). *n* = 4.

We explored the Ras/FAK-dependent signaling in SCs isolated both from Pnfs of NF1 patients (ipNF 04.4 cells) and from healthy individuals (ipNF 97.4 cells), kindly provided by Margaret Wallace (Center for Neurogenetics, University of Florida, Gainesville, FL 32608, USA). ipNF 04.4 cells displayed a high activation of ERK1/2, FAK, and Src, following stimulation with growth factors and ECM, whereas ipNF 97.4 cells underwent a lower response (Figure 4a,b), in accord with observations on MEFs. As a positive control, we used a lysate obtained from a growing Pnf (TX 1 Plex in Figure 4a,d), which showed extremely high levels of phosphorylated ERK (Figure 4a).

Most importantly, when embedded in the Matrigel matrix, ipNF 04.4 cells could form colonies, the dimension of which was strongly increased by Matrigel enrichment with Collagens/Laminin/Fibronectin, whereas 3D growth was barely detectable in ipNF 97.4 cells. Treatment with both Selumetinib and Defactinib could markedly reduce colony area (Figure 4c). To test further our hypothesis that ECM components favor neoplastic growth of Pnf SC cells, we settled on a 3D growth assay in which the medium is supplemented with insulin growth factor with or without insoluble ECM components, i.e., Laminin, Collagen I and IV, in the same proportion described in some Pnfs [44]. The aim of this experimental system is to mimic in vitro as close as possible the Pnfs’ ECM, which is typically fibrotic. The main goal was to assess whether a highly enriched collagen Matrigel (3 mg/mL), which is more rigid than a low collagen one (1 mg/mL), could affect the transforming ability of the cell. We found that, as in other cancer models [33], Matrigel enrichment with a high concentration (3 mg/mL) of Laminin and Collagen I was required for 3D growth of TX 1 primary PN cells (Figure 4e). These data clearly indicate that an abnormal ECM pilots tumor growth.

## 4. Discussion

In the present study, we addressed the still challenging issue of whether the environmental niche is determinant for *NF1* DI SC progression toward tumorigenesis and thus for neurofibroma onset. Mature Pnfs are large benign lesions whose morbidity is mainly due to the accumulation of fibrotic tissue in which *NF1* DI SCs manifest excess proliferation, displacing and even compressing adjacent tissues. A major component of the niche is the ECM, a complex network of macromolecules whose elasticity and composition determine cellular behavior [13]. Many studies have delved into the role of immune cells infiltrating neurofibromas, particularly mast cells, but little is yet known about the influence of the matrix on cellular behavior [3,6]. Here, we asked how Nfn-deficient SCs sense and perceive external signaling and whether they translate them into neoplastic transformation. We focused our attention on FAK, the multifunctional regulator of cell signaling within the microenvironment and its downstream effectors’ activity. FAK is activated by homo-dimerization and trans-phosphorylation of the main tyrosine site Y397, following either growth factor or integrin binding [15,16,17,18,19,20]. Y397 auto-phosphorylation allows FAK to function as an adapter protein that communicates with various signal transduction pathways; following cis-phosphorylation of specific phosphorylation sites, Src, Ras (through Grb 2 adaptor protein), and AKT are recruited [15]. Our results indicate that Nfn interacts with and negatively regulates FAK. In fact, in Nfn-deficient cells, we found that FAK is constitutively phosphorylated on Y397 and Y925 (binding sites of Src and Grb-2, respectively) in the absence of activating stimuli, even though cells are plated on gelatin, which minimized integrin clustering.

In cells lacking Nfn, growth factors induce higher FAK activity compared to the wild-type counterpart; similarly, Src, ERK, and AKT were found more phosphorylated, indicating their dependency on growth factors. However, this phenomenon was maximized by ECM components as Collagen or Fibronectin. In all cellular models that we exploited (MEFs, immortalized, and primary SCs from Pnfs), the raise of FAK phosphorylation correlated with increased ability to form colonies in the in vitro 3D system, suitable to assess the transforming ability of the cells.

Interestingly, spheroids were sensitive to FAK, Src, and MEK (the MAPKK upstream to ERK) inhibitors Defactinib, SU66, and Selumetinib, clearly indicating that FAK is a cross-road of these pathways. SU66 and Selumetinib, nevertheless, displayed lower selectivity than Defactinib, blocking colony growth also in wild-type cells. Cells HI for *NF1* display hyperactivation of Ras [5], which further increases when *NF1* LOH occurs [6]. Mutated K- and H-Ras are recognized to be driver mutations in several solid cancers since they hyperactivate Ras in a growth-factor-independent manner. The Ras activity generated by the loss of one of the Ras-Gaps, as it happens in the NF1 disease, is not sufficient per se to induce transformation. We, therefore, hypothesized that in Nfn-deficient cells a combination of growth factors and ECM stimuli would be able to raise the Ras activity to the threshold levels required for cell transformation [45]. Supportive of this hypothesis is the observation that ERK phosphorylation is responsive to FAK inhibitors such as Y15 and Defactinib.

However, Selumetinib alone is unable to completely abolish the colony growth of cells transfected with the catalytic domain of Nfn that restores normal Ras activity. We discovered that also AKT phosphorylation was the result of the crosstalk between Ras and FAK following either growth factor and integrin clustering. Consistently, AKT phosphorylation was decreased by cell treatment with Defactinib, clearly showing that FAK is upstream of the kinase. The role of ECM has also been investigated in other models, as human cells isolated from plexiform neurofibromas. With *NF1* LOH, cells isolated from these tumors and immortalized reproduced hyperactivity of ERK, Src partially dependent on FAK activation. The overall significance of our biochemical findings is highlighted by the fact that also these cells, such as MEFs, generated spheroids whose growth was hampered by the use of MEK and FAK inhibitors. Studying the behavior of primary cells, we realized that even after isolation, they require an abundant matrix mimicking the stiff one of the neurofibroma. In fact, in order to see colonies, we had to enrich the hydrogel (Matrigel) with abundant Collagen I.

Taken together; these lines of evidence show that the absence of Nfn activates Ras in a growth-factor-dependent manner. Nevertheless, additional signaling boosts from the matrix and are required to transform the cells making them able to grow in the absence of substrate. As a logical consequence, we reasoned that disruption of a single pathway with a single treatment would not be sufficient to disrupt this aberrant oncogenic signal, explaining the only partial clinical success of Selumetinib. Rather, the combination of both inhibitors would abolish the capacity of neoplastic growth. Instead, the combined Selumetinib and Defactinib treatment, targeting FAK, the direct mediator of the integration of ERK and AKT molecular cascades, could be the decisive therapeutic option. Our data, although in vitro, point in this direction. Given the complexity of Pnf biology, further in vitro investigation will be required by assessing the role of the focal adhesion complex in essential processes still under investigation as movement or metabolic shift [46], forcing neoplastic Schwann cells toward malignancy.

## 5. Conclusions

In this study, we show that neoplastic transformation is directed in neurofibroma cells by the cooperation of neurofibromin deficiency and extracellular matrix stimulation. Hyperactivation of focal adhesion kinase (FAK) contributes to deregulated induction of ERK and AKT downstream to Ras, conferring to Human *NF1*-deficient and mouse *Nf1*^−/^^−^ cells the ability to grow in a three-dimensional matrix. Moreover, we show that combinatorial treatment with the MEK inhibitor Selumetinib and the FAK inhibitor Defactinib fully abrogates their transforming ability, opening new therapeutic opportunities for plexiform neurofibroma treatment.

## Data Availability

All other data supporting the findings of this study can be found with the article or supplementary material.

## Supplementary Materials

The following are available online at https://www.mdpi.com/article/10.3390/cancers13102329/s1, Figure S1: Densitometry analysis of Western blotting bands expressed as median and SD of different experiments.

## References

[1] Gottfried O.N., Viskochil D.H., Couldwell W.T. (2010). Neurofibromatosis Type 1 and tumorigenesis: Molecular mechanisms and therapeutic implications. Neurosurg. Focus.

[2] Young E.D., Ingram D., Metcalf-Doetsch W., Khan D., Al Sannaa G., Le Loarer F., Lazar A.J.F., Slopis J., Torres K.E., Lev D. (2018). Clinicopathological variables of sporadic schwannomas of peripheral nerve in 291 patients and expression of biologically relevant markers. J. Neurosurg..

[3] Tucker T., Riccardi V.M., Sutcliffe M., Vielkind J., Wechsler J., Wolkenstein P., Friedman J.M. (2011). Different Patterns of Mast Cells Distinguish Diffuse from Encapsulated Neurofibromas in Patients with Neurofibromatosis 1. J. Histochem. Cytochem..

[4] Parrinello S., Lloyd A.C. (2009). Neurofibroma development in NF1—Insights into tumour initiation. Trends Cell Biol..

[5] Cichowski K., Jacks T. (2001). NF1 tumor suppressor gene function: Narrowing the GAP. Cell.

[6] Le L.Q., Parada L.F. (2007). Tumor microenvironment and neurofibromatosis type I: Connecting the GAPs. Oncogene.

[7] Gross A.M., Dombi E., Widemann B.C. (2020). Current status of MEK inhibitors in the treatment of plexiform neurofibromas. Child’s Nerv. Syst..

[8] Gross A.M., Frone M., Gripp K.W., Gelb B.D., Schoyer L., Schill L., Stronach B., Biesecker L.G., Esposito D., Hernandez E.R. (2020). Advancing RAS/RASopathy therapies: An NCI-sponsored intramural and extramural collaboration for the study of RASopathies. Am. J. Med. Genet. Part A.

[9] Gross A.M., Widemann B.C. (2020). Clinical trial design in neurofibromatosis type 1 as a model for other tumor predisposition syndromes. Neuro-Oncol. Adv..

[10] Gross A.M., Wolters P.L., Dombi E., Baldwin A., Whitcomb P., Fisher M.J., Weiss B., Kim A., Bornhorst M., Shah A.C. (2020). Selumetinib in Children with Inoperable Plexiform Neurofibromas. N. Engl. J. Med..

[11] Harrisingh M.C., Lloyd A.C. (2004). Ras/Raf/ERK signalling and NF1. Cell Cycle.

[12] Aragona M., Panciera T., Manfrin A., Giulitti S., Michielin F., Elvassore N., Dupont S., Piccolo S. (2013). A Mechanical Checkpoint Controls Multicellular Growth through YAP/TAZ Regulation by Actin-Processing Factors. Cell.

[13] Northey J.J., Przybyla L., Weaver V.M. (2017). Tissue Force Programs Cell Fate and Tumor Aggression. Cancer Discov..

[14] Ratner N., Miller S.J. (2015). A RASopathy gene commonly mutated in cancer: The neurofibromatosis type 1 tumour suppressor. Nat. Rev. Cancer.

[15] Mitra S.K., Hanson D.A., Schlaepfer D.D. (2005). Focal adhesion kinase: In command and control of cell motility. Nat. Rev. Mol. Cell Biol..

[16] Zhao J., Guan J.L. (2009). Signal transduction by focal adhesion kinase in cancer. Cancer Metastasis Rev..

[17] Zhu J., Wang Y.-S., Zhang J., Zhao W., Yang X.-M., Li X., Jiang T.-S., Yao L.-B. (2009). Focal adhesion kinase signaling pathway participates in the formation of choroidal neovascularization and regulates the proliferation and migration of choroidal microvascular endothelial cells by acting through HIF-1 and VEGF expression in RPE cells. Exp. Eye Res..

[18] Hauck C.R., Hsia D.A., Ilic D., Schlaepfer D.D. (2002). v-Src SH3-enhanced interaction with focal adhesion kinase at beta 1 integrin-containing invadopodia promotes cell invasion. J. Biol. Chem..

[19] Hauck C.R., Hsia D.A., Puente X.S., Cheresh D.A., Schlaepfer D.D. (2002). FRNK blocks v-Src-stimulated invasion and experimental metastases without effects on cell motility or growth. EMBO J..

[20] Hauck C.R., Hsia D.A., Schlaepfer D.D. (2000). Focal Adhesion Kinase Facilitates Platelet-derived Growth Factor-BB-stimulated ERK2 Activation Required for Chemotaxis Migration of Vascular Smooth Muscle Cells. J. Biol. Chem..

[21] Hauck C.R., Hsia D.A., Schlaepfer D.D. (2002). The Focal Adhesion Kinase--A Regulator of Cell Migration and Invasion. IUBMB Life.

[22] Hauck C.R., Hunter T., Schlaepfer D.D. (2001). The v-Src SH3 Domain Facilitates a Cell Adhesion-independent Association with Focal Adhesion Kinase. J. Biol. Chem..

[23] Goode E.L., Chenevix-Trench G., Song H., Ramus S.J., Notaridou M., Lawrenson K., Widschwendter M., Vierkant R.A., Larson M.C., Kjaer S.K. (2010). A genome-wide association study identifies susceptibility loci for ovarian cancer at 2q31 and 8q24. Nat. Genet..

[24] Grove M., Komiyama N.H., Nave K.-A., Grant S.G., Sherman D.L., Brophy P.J. (2007). FAK is required for axonal sorting by Schwann cells. J. Cell Biol..

[25] Zhang K., Jiang M.N., Zhang C.H., Li C., Jia Y.J. (2014). Effects of Ganfukang on expression of connective tissue growth factor and focal adhesion kinase/protein kinase B signal pathway in hepatic fibrosis rats. Chin. J. Integr. Med..

[26] Chen J.-S., Li H.-S., Huang J.-Q., Dong S.-H., Huang Z.-J., Yi W., Zhan G.-F., Feng J.-T., Sun J.-C., Huang X.-H. (2016). MicroRNA-379-5p inhibits tumor invasion and metastasis by targeting FAK/AKT signaling in hepatocellular carcinoma. Cancer Lett..

[27] Sulzmaier F.J., Jean C., Schlaepfer D.D. (2014). FAK in cancer: Mechanistic findings and clinical applications. Nat. Rev. Cancer.

[28] Mohanty A., Pharaon R.R., Nam A., Salgia S., Kulkarni P., Massarelli E. (2020). FAK-targeted and combination therapies for the treatment of cancer: An overview of phase I and II clinical trials. Expert Opin. Investig. Drugs.

[29] O’Brien S., Golubovskaya V.M., Conroy J., Liu S., Wang D., Liu B., Cance W.G. (2014). FAK inhibition with small molecule inhibitor Y15 decreases viability, clonogenicity, and cell attachment in thyroid cancer cell lines and synergizes with targeted therapeutics. Oncotarget.

[30] Marusak C., Thakur V., Li Y., Freitas J.T., Zmina P.M., Thakur V.S., Chang M., Gao M., Tan J., Xiao M. (2020). Targeting Extracellular Matrix Remodeling Restores BRAF Inhibitor Sensitivity in BRAFi-resistant Melanoma. Clin. Cancer Res..

[31] Zhang L., Zhao D., Wang Y., Zhang W., Zhang J., Fan J., Zhan Q., Chen J. (2021). Focal adhesion kinase (FAK) inhibitor-defactinib suppresses the malignant progression of human esophageal squamous cell carcinoma (ESCC) cells via effective blockade of PI3K/AKT axis and downstream molecular network. Mol. Carcinog..

[32] Canel M., Serrels A., Miller D., Timpson P., Serrels B., Frame M.C., Brunton V.G. (2010). Quantitative In vivo Imaging of the Effects of Inhibiting Integrin Signaling via Src and FAK on Cancer Cell Movement: Effects on E-cadherin Dynamics. Cancer Res..

[33] Jiang H., Hegde S., Knolhoff B.L., Zhu Y., Herndon J.M., Meyer M.A., Nywening T.M., Hawkins T.M.N.W.G., Shapiro I.M., Weaver D.T. (2016). Targeting focal adhesion kinase renders pancreatic cancers responsive to checkpoint immunotherapy. Nat. Med..

[34] Gerber D.E., Camidge D.R., Morgensztern D., Cetnar J., Kelly R.J., Ramalingam S.S., Spigel D.R., Jeong W., Scaglioni P.P., Zhang S. (2020). Phase 2 study of the focal adhesion kinase inhibitor defactinib (VS-6063) in previously treated advanced KRAS mutant non-small cell lung cancer. Lung Cancer.

[35] Tavora B., Reynolds L.E., Batista S., Demircioglu F., Fernandez I., Lechertier T., Lees D.M., Wong P.-P., Alexopoulou A., Elia G. (2014). Endothelial-cell FAK targeting sensitizes tumours to DNA-damaging therapy. Nature.

[36] Li H., Chang L.-J., Neubauer D.R., Muir D.F., Wallace M.R. (2016). Immortalization of human normal and NF1 neurofibroma Schwann cells. Lab. Investig..

[37] Shapira S.D., Barkan B., Friedman E., Kloog Y., Stein R.E. (2006). The tumor suppressor neurofibromin confers sensitivity to apoptosis by Ras-dependent and Ras-independent pathways. Cell Death Differ..

[38] Chiara F., Bishayee S., Heldin C.H., Demoulin J.B. (2004). Autoinhibition of the platelet-derived growth factor beta-receptor tyrosine kinase by its C-terminal tail. J. Biol. Chem..

[39] Chiara F., Goumans M.J., Forsberg H., Ahgren A., Rasola A., Aspenstrom P., Wernstedt C., Hellberg C., Heldin C.H., Heuchel R. (2004). A gain of function mutation in the activation loop of platelet-derived growth factor beta-receptor deregulates its kinase activity. J. Biol. Chem..

[40] Dixon R.D., Chen Y., Ding F., Khare S.D., Prutzman K.C., Schaller M.D., Campbell S.L., Dokholyan N.V. (2004). New Insights into FAK Signaling and Localization Based on Detection of a FAT Domain Folding Intermediate. Structure.

[41] Golubovskaya V., Curtin L., Groman A., Sexton S., Cance W.G. (2014). In vivo toxicity, metabolism and pharmacokinetic properties of FAK inhibitor 14 or Y15 (1, 2, 4, 5-benzenetetramine tetrahydrochloride). Arch. Toxicol..

[42] Gale N.W., Kaplan S., Lowenstein E.J., Schlessinger J., Bar-Sagi D. (1993). Grb2 mediates the EGF-dependent activation of guanine nucleotide exchange on Ras. Nature.

[43] Schlaepfer D.D., Hauck C.R., Sieg D.J. (1999). Signaling through focal adhesion kinase. Prog. Biophys. Mol. Biol..

[44] Guarino M. (1993). Plexiform schwannoma. Immunohistochemistry of Schwann cell markers, intermediate filaments and extracellular matrix components. Pathol. Res. Pract..

[45] Zinatizadeh M.R., Momeni S.A., Zarandi P.K., Chalbatani G.M., Dana H., Mirzaei H.R., Akbari M.E., Miri S.R. (2019). The Role and Function of Ras-association domain family in Cancer: A Review. Genes Dis..

[46] Masgras I., Ciscato F., Brunati A.M., Tibaldi E., Indraccolo S., Curtarello M., Chiara F., Cannino G., Papaleo E., Lambrughi M. (2017). Absence of Neurofibromin Induces an Oncogenic Metabolic Switch via Mitochondrial ERK-Mediated Phosphorylation of the Chaperone TRAP1. Cell Rep..

