# Supplementary material for: Neurofibromin Deficiency and Extracellular Matrix Cooperate to Increase Transforming Potential through FAK-Dependent Signaling
Source: Cancers (Basel). 2021 May 12;13(10):2329. doi: 10.3390/cancers13102329 (PMC8150846; doi:10.3390/cancers13102329)
Supplement: Supplementary file 1 [file cancers-13-02329-s001.zip › cancers-1145770-supplementary.pdf]

cancers

MDPI

Supplementary Materials

Neurofibromin Deficiency and Extracellular Matrix Cooperate to Increase Transforming Potential Through FAK-Dependent Signalling

Andrea Errico et al.

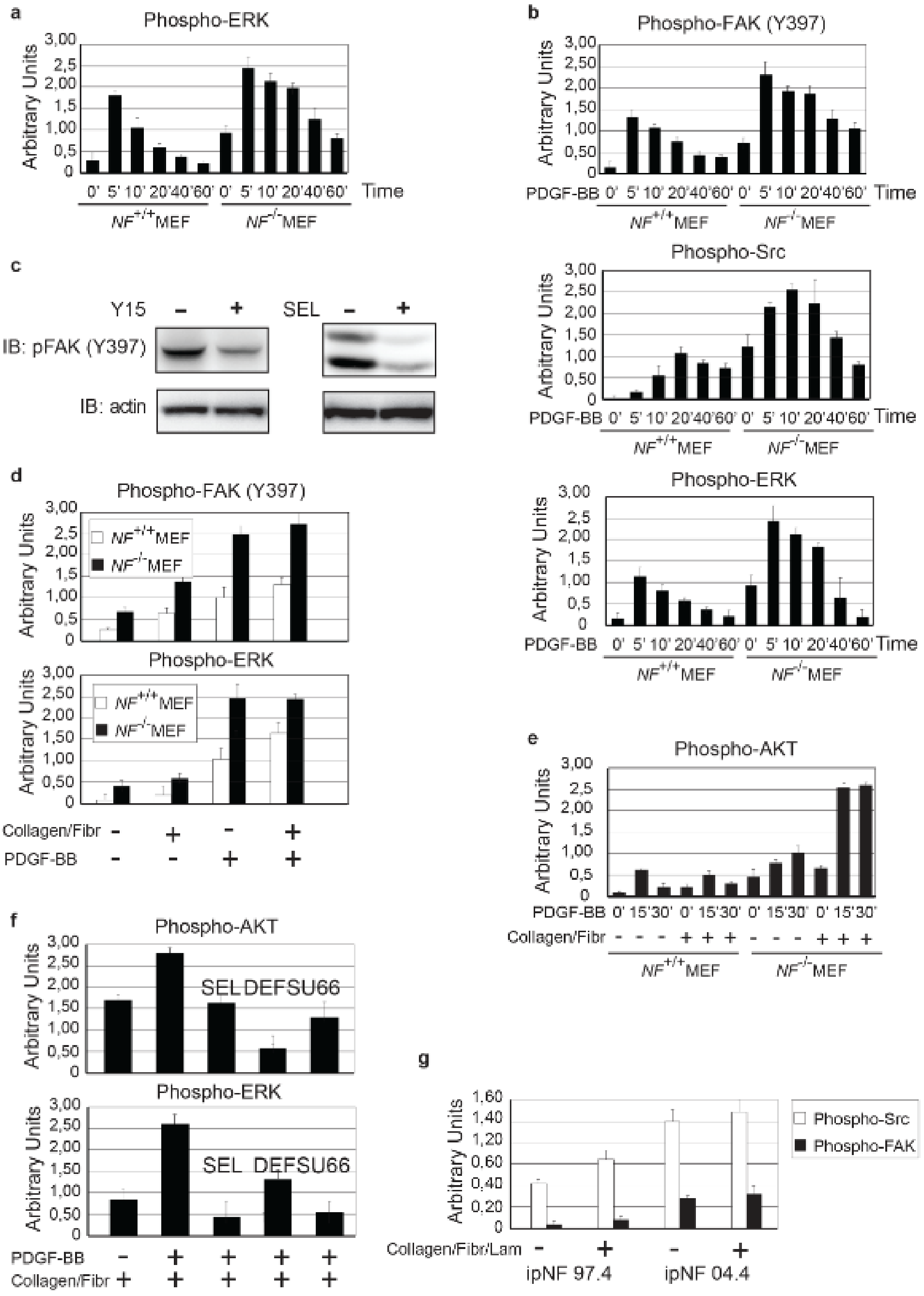

Figure S1. Densitometry analysis of western blotting bands expressed as median and SD of different experiments. (**a**) Densitometry analysis of phosphorylation kinetics of ERK (1/2) in *Nf1* MEFs $n = 4$. (see Figure 1) (**b**) Densitometry analysis

of phosphorylation kinetics of ERK (1/2), FAK and Src kinases in *Nf1* MEFs *n* = 5. (**c**) Western blotting showing the effectiveness of Y15 and Selumetinib inhibitor treatment on $Nf1^{-/-}$ MEFs. (**d**–**e**) Densitometry analysis of phosphorylation levels of ERK (1/2), FAK, Src, and AKT kinases in *Nf1* MEFs under Collagen/Fibronectin stimulation (see Figure 2) *n* = 3. (**f**) Densitometry analysis of phosphorylation levels of ERK (1/2), and AKT kinases in colonies of *Nf1* MEFs grown in *3D* in vitro under Growth factor and Collagen/Fibronectin stimulation in presence or absence of different kinase inhibitors (see Figure 3) *n* = 4. (**g**) Densitometry analysis of phosphorylation levels of ERK (1/2), FAK, Src, and AKT kinases in Schwann cells ipNF 97.4 and 04.4 ($Nf1^{+/+}$, $Nf1^{-/-}$, respectively) under Collagen/Fibronectin/Laminin stimulation (see Figure 2) *n* = 5.
